# Association of serum 25-hydroxyvitamin D levels with age-related macular degeneration and its clinical correlates: a cross-sectional study

**DOI:** 10.3389/fendo.2025.1635739

**Published:** 2025-08-14

**Authors:** Xu Liang, Jiaxing Wang, Yue Zhang, Hui Zheng

**Affiliations:** ^1^ Tianjin Eye Hospital, Tianjin Key Lab of Ophthalmology and Visual Science, Tianjin Eye Institute, Tianjin, China; ^2^ Bascom Palmer Eye Institute, University of Miami, Miami, FL, United States

**Keywords:** age-related macular degeneration (AMD), 25-hydroxyvitamin D, calcium homeostasis, biomarker, vitamin D deficiency

## Abstract

**Introduction:**

Age-related macular degeneration (AMD) is a leading cause of irreversible vision loss in older adults, with significant inter-individual variability in clinical progression. Vitamin D, known for its role in calcium homeostasis and anti-inflammatory pathways, may be implicated in AMD pathogenesis. This study aimed to investigate serum 25-hydroxyvitamin D [25(OH)D] levels in AMD patients and their association with clinical phenotypes.

**Methods:**

This single-center, cross-sectional observational study was conducted at Tianjin Eye Hospital, China, involving 210 participants (100 AMD patients and 110 healthy controls). Exclusion criteria included conditions affecting vitamin D metabolism and recent vitamin D supplementation. Comprehensive ophthalmic assessments and laboratory tests were performed. Data were analyzed using R software, employing Student’s t-tests, ANONA, chi-squared tests, Pearson correlation and linear regression models.

**Results:**

AMD patients exhibited significantly lower serum 25(OH)D levels than controls (22.98 ± 7.30 ng/mL vs. 26.12 ± 9.81 ng/mL, p=0.013). Within the AMD group, late-stage patients had lower 25(OH)D levels than early-stage patients (22.53 ± 8.14 ng/mL vs. 23.46 ± 6.36 ng/mL, p=0.019) and higher CRP levels (0.31 ± 0.19 mg/L vs. 0.17 ± 0.05 mg/L, p=0.015). ROC curve analysis indicated moderate diagnostic utility of 25(OH)D for distinguishing AMD patients from controls (AUC=0.714, 95% CI: 0.58-0.73, p<0.01), but limited ability to differentiate early vs. late-stage AMD Linear regression analysis revealed positive associations between 25(OH)D levels and apolipoprotein E (ApoE, β=0.157, p=0.04) and serum creatinine (β=0.18, p=0.02).

**Conclusion:**

This study provides evidence linking lower serum 25(OH)D levels to the presence and severity of AMD, particularly in late-stage disease.

## Introduction

Age-related macular degeneration (AMD), the leading cause of irreversible vision loss in older adults, is characterized by heterogeneous progression-from early drusen formation and retinal pigment epithelium (RPE) dysfunction to advanced stages of geographic atrophy (GA) or choroidal neovascularization (CNV) (1). The clinical progression of AMD varies significantly across individuals, with notable differences in both the severity of the disease and its temporal course (1). Importantly, while many patients maintain stable disease with only drusenoid changes that preserve functional visual acuity, others may face progressive RPE degeneration due to GA expansion or experience sudden vision loss resulting from the development of exudative CNV. Established modifiable risk determinants for age-related macular degeneration (AMD) development and progression encompass cigarette smoking, hypertension, abdominal adiposity, physical inactivity, and dietary lipid consumption (2–4). Clinical evidence demonstrates that pharmacological intervention with high-dose antioxidant formulations, alongside emerging therapeutic strategies targeting macular carotenoid supplementation, effectively attenuates the transition from intermediate nonneovascular AMD (NNVAMD) to advanced disease phenotypes (5). Given the well-characterized involvement of inflammatory signaling and pathological angiogenesis in AMD pathogenesis, physiological or environmental factors influencing these biological processes may modulate the progression from NNVAMD to neovascular AMD (NVAMD).

Vitamin D is recognized as a potential protective factor against a variety of diseases, including cancer, cardiovascular conditions, bone disorders, kidney diseases, and others (6). Calcium homeostasis is a crucial process for various cellular and biological functions, primarily regulated by vitamin D (7). Emerging evidence implicates calcium homeostasis dysregulation in AMD pathophysiology, particularly through mechanisms involving aging processes and drusen formation (8, 9). Mechanistic investigations reveal that RPE cells in advanced AMD exhibit concurrent autophagy impairment and inflammasome hyperactivation, with corresponding depletion of intracellular free Ca²^+^ stores—a pathophysiological signature suggesting calcium signaling deficits may drive AMD progression (10). Clinical correlations further identify hydroxyapatite deposition within macular lesions as a potential radiographic biomarker predictive of late-stage AMD conversion (11). Beyond its systemic effects, emerging evidence suggests vitamin D may play a role in ocular diseases, including AMD, through its regulation of calcium homeostasis and anti-inflammatory pathways.

Building on vitamin D’s role in calcium homeostasis, research has investigated its impact on AMD. Epidemiological analyses present paradoxical associations: Research has indicated that higher serum levels of 25(OH)D are linked to an increased incidence of early AMD, while also being associated with a decreased incidence of late-stage AMD (12). Previous studies have demonstrated that elevated vitamin D levels and dietary intake are correlated with a lower likelihood of developing AMD (13, 14). However, a recent systematic review and meta-analysis encompassing 16 studies found no definitive evidence supporting a clear association between serum 25(OH)D levels and the risk of AMD (15). Similarly, population-based studies demonstrate protective effects of dietary calcium supplementation against AMD incidence (16), whereas contradictory reports paradoxically link calcium supplementation to increased AMD prevalence in specific cohorts (17). These divergent findings highlight significant uncertainties regarding the therapeutic implications of calcium modulation and its interplay with vitamin D metabolism in AMD pathogenesis across disease subtypes.

To address these knowledge gaps, the aim of the present study was to determine 25(OH)D levels in AMD patients, as well as relevant contributing factors.

## Method

This was a single-center, cross-sectional observational study conducted at the Tianjin Eye Hospital, China, designed to characterize serum 25(OH)D levels in patients with AMD and explore associations with clinical phenotypes. The study adhered to the Declaration of Helsinki and was approved by the institutional review board (IRB No. 2020048), with written informed consent obtained from all participants.

### Participants and exclusion criteria

Participants aged ≥50 years were recruited between January 2022 and December 2023. Exclusion criteria included: (i) history of glaucoma, iris neovascularization, ocular trauma, uveitis, intraocular injection, vitrectomy, or other vitreoretinal diseases;(ii) systemic conditions affecting vitamin D metabolism, such as osteoporosis, chronic renal failure, liver disease, or parathyroid dysfunction (confirmed via medical records or patient interviews);(iii) use of vitamin D supplements (≥400 IU/day) within 3 months prior to enrollment. Patients underwent assessments of serum 25(OH)D, serum lipoproteins, renal function, serum apolipoprotein E (ApoE). All participants completed the questionnaire survey, those unable to complete it independently were assisted by a middle-aged companion during the process.

### Ophthalmic assessments and AMD staging

Comprehensive eye examinations included:

Best-corrected visual acuity (BCVA) measured at 4 meters using a Snellen chart, converted to logMAR units for analysis;Anterior segment evaluation via slit-lamp biomicroscopy and intraocular pressure measurement by applanation tonometry;Fundus imaging with color photography and spectral-domain optical coherence tomography (SD-OCT, Zeiss 3D OCT-2000) to identify drusen, retinal pigment epithelium changes, and neovascular lesions. AMD was classified into early-stage (small/medium drusen or RPE alterations) and late-stage (large drusen, geographic atrophy, or choroidal neovascularization) according to the Ferris clinical criteria (18).

### Sample testing

All samples were collected and tested in the same period as much as possible to avoid deviations caused by different seasons. 25(OH)D was determined by enzyme - linked immunosorbent assay (ELISA) (Roche Diagnostics).

### Statistical analysis

Data were analyzed using R software (Version 4.2.1, https://www.r-project.org/). Normality was tested via the Kolmogorov-Smirnov test. Parametric tests (Student’s t-test, ANOVA) were used for normally distributed data, while non-parametric tests (Kruskal-Wallis, H) were applied to skewed variables. Categorical data were compared using chi-squared tests. Receiver operating characteristic (ROC) curves evaluated the diagnostic utility of 25(OH)D. Peasrson correlation and linear regression models explored associations with clinical variables, adjusting for confounders. A p-value < 0.05 indicated statistical significance.

## Result

### Data characteristics

A total of 210 participants were enrolled, including 110 healthy controls and 100 AMD patients. Using the Ferris clinical classification, 50 AMD patients were categorized as early-stage, and 50 as late-stage. The late-stage subgroup included 50 cases of nAMD. Baseline demographics are presented in **Tables 1** and **2**, showing no significant differences in age, sex, body mass index (BMI), or education between groups (all *p* > 0.05).

**Table 1:** Main characteristics of patients included in this study.

Characteristics	AMD(100)	control(110)	t/χ²	*p*
age(mean±SD)	66.22±6.39	67.48±8.60		0.94
gender(%)				
famale	65.7	65.9	0.33	0.89
Education(%)				
No Formal Education	5.2	1	7.36	0.12
Primary School	4.2	12.4		
Junior High School	32.3	28.6		
Senior High School	31.3	29.5		
College/University or Above	27.1	28.6		
BMI(mean±SD)	24.79±3.23	24.59±3.29	0.44	0.92
25(OH)D (mean±SD)	22.98±7.30	26.12±9.81	2.613	0.013
CRP(mean±SD)	0.24±0.39	0.2±0.19	-1.05	0.048
GLU(mean±SD)	4.76±0.89	4.9±0.96	1.09	0.86
TG(mean±SD)	1.49±0.79	1.74±1.50	1.46	0.12
TC(mean±SD)	5.26±0.94	5.49±1.11	1.59	0.23
LDLC(mean±SD)	3.17±0.91	3.22±0.82	0.42	0.33
HDLC(mean±SD)	1.36±0.27	1.43±3.23	1.56	0.22
APOE(mean±SD)	48.52±15.27	48.97±17.13	0.20	0.62
UREA(mean±SD)	5.96±1.43	6.10±1.64	0.64	0.45
CREA(mean±SD)	66.69±15.46	65.46±14.51	-0.60	0.32

Values are presented as mean ± standard deviation. BMI, Body Mass Index; 25 (OH)D, 25-Hydroxyvitamin D; CRP, C-Reactive Protein; GLU, Glucose; TG, Triglyceride; TC, Total Cholesterol; LDLC, Low-Density Lipoprotein Cholesterol; HDLC, High-Density Lipoprotein Cholesterol; APOE, Apolipoprotein E; CREA, Creatinine.

**Table 2:** Main characteristics of patients in AMD group.

Characteristics	Early-AMD(50)	Late-AMD(50)	t/χ²	*p*
age(mean±SD)	67.38±6.56	67.11±6.64	-0.21	0.43
gender(%)				
famale	73.1	58.5	2.47	0.11
Education(%)				
No Formal Education	5.8	4.5	5.14	0.27
Primary School	5.8	2.3		
Junior High School	23.1	43.2		
Senior High School	32.7	29.5		
College/University or Above	32.7	20.5		
BMI(mean±SD)	24.19±2.90	25.26±3.70	1.64	0.13
25(OH)D (mean±SD)	23.46±6.36	22.53±8.14	1.71	0.019
CRP(mean±SD)	0.17±0.05	0.31±0.19	1.74	0.015
GLU(mean±SD)	4.75±0.64	4.77±1.06	0.11	0.086
TG(mean±SD)	1.41±0.69	1.77±1.81	1.35	0.17
TC(mean±SD)	5.31±0.92	5.21±0.95	-0.55	0.59
LDLC(mean±SD)	3.27±0.78	3.07±0.80	-1.27	0.85
HDLC(mean±SD)	1.35±0.28	1.36±0.27	0.16	0.75
APOE(mean±SD)	47.92±13.81	49.32±16.56	0.47	0.62
UREA(mean±SD)	5.84±1.53	6.09±1.28	0.35	0.88
CREA(mean±SD)	64.33±15.69	68.73±14.77	1.48	0.99

Values are presented as mean ± standard deviation. BMI, Body Mass Index; 25 (OH)D, 25-Hydroxyvitamin D; CRP, C-Reactive Protein; GLU, Glucose; TG, Triglyceride; TC, Total Cholesterol; LDLC, Low-Density Lipoprotein Cholesterol; HDLC, High-Density Lipoprotein Cholesterol; APOE, Apolipoprotein E; CREA, Creatinine.

AMD patients had significantly lower serum 25(OH)D levels than controls (22.98 ± 7.30 vs. 26.12 ± 9.81 ng/mL, t=2.613, p=0.013, **Table 1**), and significantly higher CRP(0.24 ± 0.39 vs. 0.2 ± 0.19mg/L, t=-1.05, p= 0.048, **Table 1**). Within the AMD group, late-stage patients exhibited lower 25(OH)D levels than early-stage patients (22.53 ± 8.14 vs. 23.46 ± 6.36 ng/mL, t = 1.71, *p=* 0.019) and higher C-reactive protein (CRP) levels (0.31 ± 0.19 vs. 0.17 ± 0.05 mg/L, t = 1.74, *p=* 0.015, **Table 2**), despite similar baseline characteristics.

### Diagnostic performance

**Figure 1:**
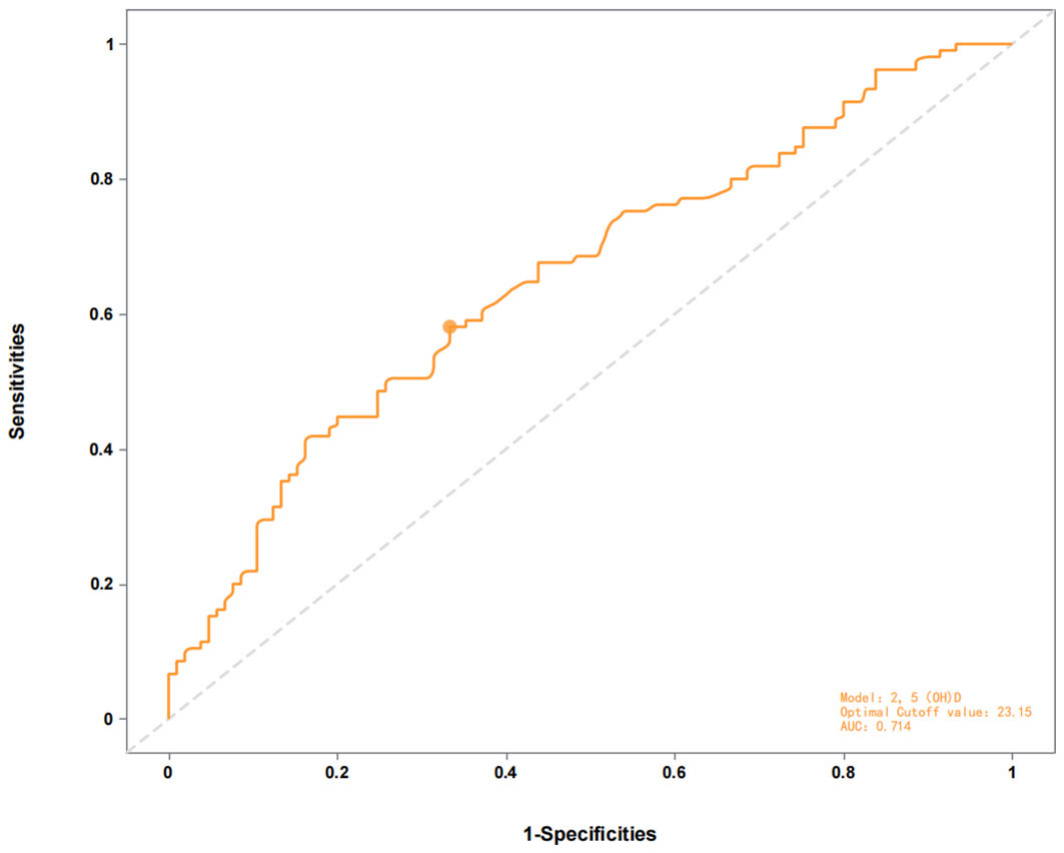
ROC curve of the 2,5(0H)D model for distinguishing AMD patients from controls (AUC = 0.714) with optimal cutoff at 23.15.

**Figure 2:**
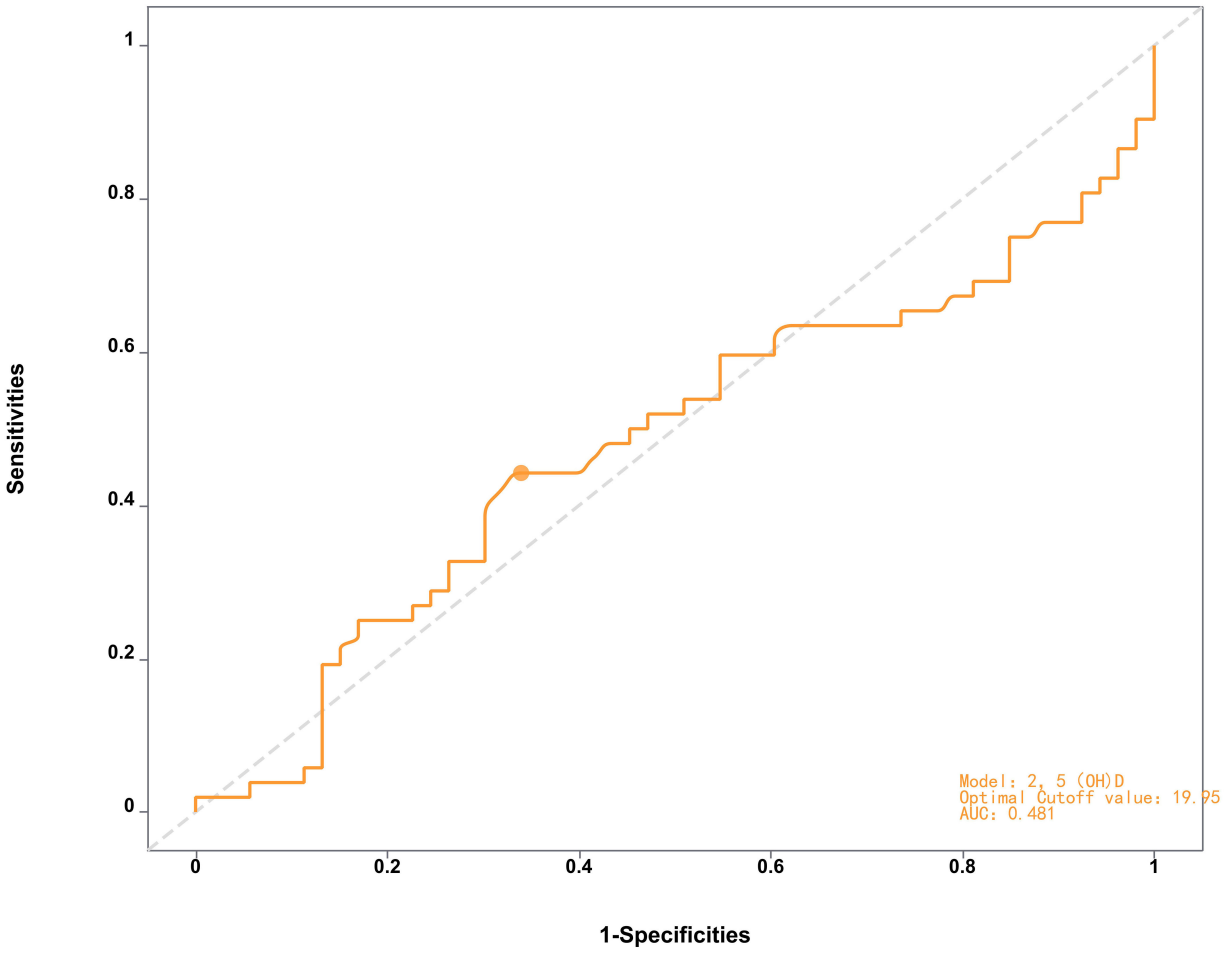
ROC curve of the 2,5(QHD) model distinguishing late-AMD patients from early-AMD indicating limited diagnostic performance (AUC = 0.481) with optimal cutoff at 19.95.

ROC curve analysis revealed moderate discriminative power of 25(OH)D for distinguishing AMD patients from controls (area under the curve [AUC] = 0.714, 95% CI: 0.58–0.73, *p<* 0.01, **Figure 1**, **Table 3**), with an optimal threshold of 23.15 ng/mL. In contrast, 25(OH)D did not significantly differentiate early vs. late-stage AMD (AUC = 0.481, 95% CI: 0.41–0.63, *p=* 0.058, **Figure 2**).

**Table 3:** Predictive performance of the evaluated models.

Model	AUC	cut - off	*p*	95%CI
AMD/control	0.714	23.15	<0.01	(0.58 - 0.73)
early/late	0.481	19.95	0.058	(0.41 - 0.63)

### Correlates of 25(OH)D levels

**Figure 3:**
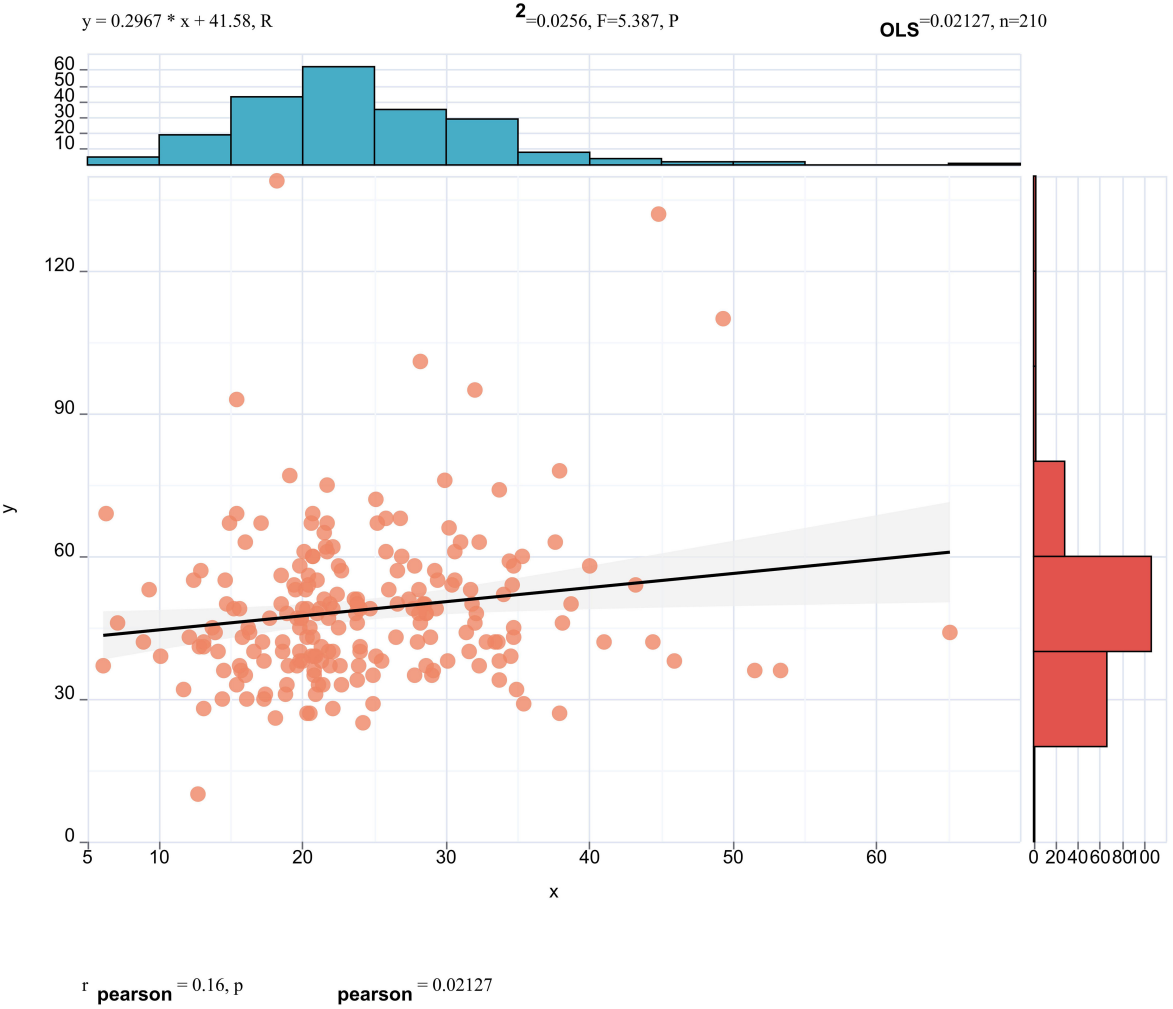
Pearson correlation analysis revealed that serum ApoE (correlation coefficient = 0.16, p < 0.05) were significantly correlated with 25(OH)D] levels.

**Figure 4:**
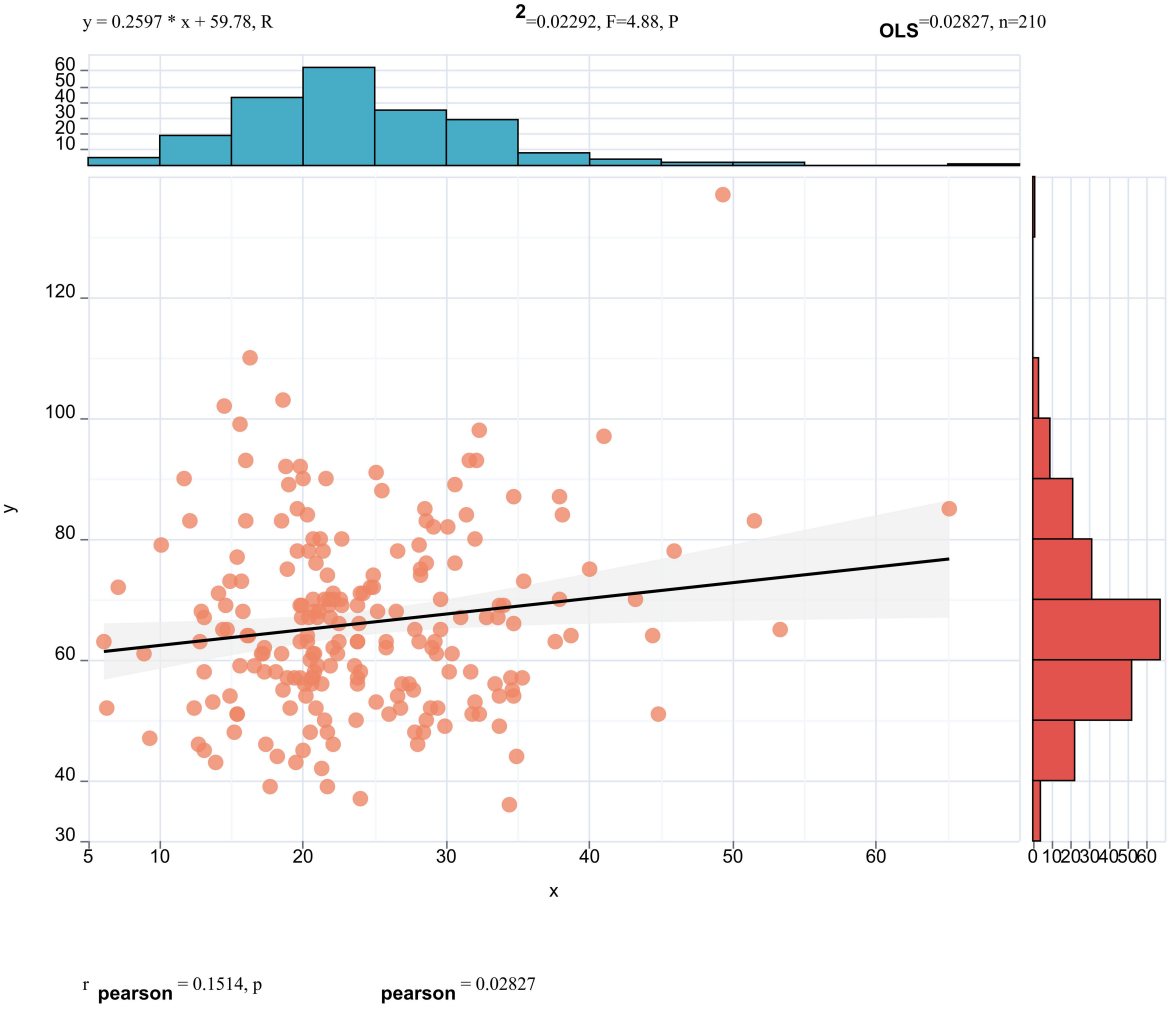
Pearson correlation analysis revealed that serum CREA (correlation coefficient = 0.15, p < 0.05) were significantly correlated with 25(OH)D] levels.

Pearson correlation analysis revealed that serum ApoE (correlation coefficient = 0.16, *p* < 0.05, **Figure 3**) and CR(correlation coefficient = 0.15, *p*< 0.05, **Figure 4**) were significantly correlated with25(OH)D] levels. Adjusted for age, sex, education level and BMI, multiple linear regression analysis demonstrated significant positive associations of serum 25(OH)D levels with apolipoprotein E (ApoE; β = 0.157, *p* = 0.04) and serum CR(β = 0.18, *p* = 0.02), while revealing a negative association with age (β = −0.14, *p* = 0.043) (**Table 4**).

**Table 4:** Linear regression analysis of factors associated with serum 25(OH)D levels.

var	β	p
BMI	0.006	0.946
CRP	-0.14	0.068
GLU	0.04	0.62
TG	-0.081	0.26
TC	0.20	0.24
HDLC	-0.12	0.16
LDLC	-0.12	0.42
ApoE	0.16	0.041
CREA	0.18	0.021
UREA	-0.038	0.61
age	-0.14	0.043

BMI, Body Mass Index; 25(OH)D, 25-Hydroxyvitamin D; CRP, C-Reactive Protein; GLU, Glucose; TG, Triglyceride; TC, Total Cholesterol; LDLC, Low-Density Lipoprotein Cholesterol; HDLC, High-Density Lipoprotein Cholesterol; APOE, Apolipoprotein E; CREA, Creatinine; p<0.05.

## Discussion

This study demonstrated that serum 25(OH)D levels are significantly lower in AMD patients than in healthy controls, with moderate diagnostic utility for distinguishing AMD (AUC = 0.714). Subgroup analysis further revealed that late-stage AMD patients exhibit lower 25(OH)D levels than early-stage patients, suggesting a potential role for vitamin D deficiency in disease progression despite the modest absolute difference. This finding aligns with the gradient of decreasing vitamin D levels alongside increasing disease severity, and may be interpreted in conjunction with the concurrent increase in CRP levels, implying a link to inflammatory pathways. Notably, late-stage AMD patients exhibited lower 25(OH)D levels than early-stage patients, aligning with the hypothesis that vitamin D deficiency may contribute to disease progression. Our findings partially reconcile prior conflicting evidence. A meta-analysis in 2016 indicated that age-related reductions in 25(OH)D levels may increase susceptibility to AMD onset and progression, which aligns with our results (19). In contrast, another meta-analysis failed to demonstrate a significant inverse relationship between serum vitamin D levels and AMD risk, possibly due to differences in study populations and methods (20). Notably, a 2021 meta-analysis also found no clear association between 25(OH)D levels and AMD incidence but observed a trend towards progression to wet AMD in individuals with low 25(OH)D concentrations (21). Our study further supports the hypothesis that vitamin D deficiency may contribute to disease progression and extends these findings by demonstrating a significant difference in 25(OH)D levels between early and late-stage AMD patients.

Oxidative stress represents a key pathogenic determinant in the etiopathogenesis of macular degeneration, characterized by intracellular macromolecular damage induced by reactive oxygen species (ROS)—including superoxide anions, hydroxyl radicals, and hydrogen peroxide. This biochemical imbalance instigates and propagates chronic inflammatory cascades via prolonged, excessive, or dysregulated oxidative stress burden, a pathophysiological mechanism previously implicated in the progression of both neovascular (nAMD) and geographic atrophy (GA) phenotypes of advanced age-related macular degeneration (AMD) (22–24). Such redox dyshomeostasis perpetuates retinal pigment epithelial (RPE) dysfunction and choroidal neovascularization, underscoring its role in the degenerative cascade underlying end-stage AMD phenotypes. Vitamin D is postulated to exert a pivotal role in ocular pathobiology, supported by preclinical evidence demonstrating expression of the vitamin D receptor (VDR) and its metabolic enzymes—cytochrome P450 family 27 subfamily B member 1 (CYP27B1) and cytochrome P450 family 24 subfamily A member 1 (CYP24A1)—within ocular tissues, including the retina, retinal pigment epithelium (RPE), and choroid (13). Furthermore, the immunomodulatory properties of vitamin D in intraocular inflammatory conditions have been well characterized (25), underscoring its potential mechanistic relevance to ocular disease pathogenesis. A 2024 *in vitro* study using the ARPE-19 cell line further demonstrated that vitamin D supplementation markedly reduced pro-inflammatory responses in RPE cells. Experimental analyses demonstrated that vitamin D supplementation significantly attenuated pro-inflammatory responses in RPE cells, corroborating the hormone’s beneficial role in modulating inflammatory pathways relevant to ocular pathobiology (26). Additionally, this study aligns with prior research showing that the vitamin D/vitamin D receptor (VDR) axis exerts negative feedback regulation on transforming growth factor-β (TGF-β) signaling (26). The present findings extend these observations by confirming vitamin D’s broader function in counteracting cytokine-mediated pro-inflammatory activity, thereby highlighting its potential as a modulator of RPE cell homeostasis. Collectively, these data provide mechanistic insights into vitamin D’s anti-inflammatory actions in ocular tissues, supporting further exploration of its therapeutic implications for inflammatory retinal disorders.

In this study, 25(OH)D served as the biomarker for vitamin D status, reflecting both dietary intake and cutaneous synthesis as the precursor to 1,25-dihydroxyvitamin D [1,25(OH)_2_D]. As the established clinical gold standard, 25(OH)D is widely recognized for evaluating systemic vitamin D levels due to its role in reflecting both dietary intake and cutaneous synthesis, ensuring reliable and standardized assessments in research and clinical practice. Given the established dependence of 25(OH)D systemic metabolism and bioavailability on hepatic and renal functional integrity, our study demonstrated statistically significant associations between serum 25(OH)D apolipoprotein E (ApoE), and creatinine (CR). The relationship between 25(OH)D and ApoE is less well-defined, with limited direct evidence and mostly indirect associations via lipid metabolism. Apolipoproteins are closely associated with the levels of serum lipoproteins. This study is the first to directly reveal the relationship between ApoE and vitamin D through clinical research. While the positive correlation between serum ApoE and 25(OH)D (β=0.157, p=0.04) suggests a novel association, this finding requires validation in mechanistic studies. The link may involve lipid metabolism or shared genetic pathways, but causal relationships—such as ApoE isoform-specific effects on vitamin D homeostasis—warrant further investigation. Previous studies have reported inconsistent findings regarding the relationship between serum apolipoprotein E (ApoE) levels and low-density lipoprotein (LDL) levels (27–31). This variability may stem from differences in study design, population characteristics (e.g., age, ethnicity, or health status), ApoE genetic polymorphisms (e.g., ϵ2, ϵ3, ϵ4 alleles), or methodological factors such as assay specificity and statistical adjustments for confounders. For instance, some studies suggest that ApoE isoforms differentially regulate lipid metabolism, with the ϵ4 allele potentially promoting LDL retention, while ϵ2 may reduce LDL clearance (29–31). Additionally, interactions with environmental factors (e.g., diet, comorbidities) or analytical approaches (e.g., inclusion of covariates like HDL or triglycerides) could further contribute to divergent results. However, our study results indicate no significant association between LDL/HDL levels and 25(OH)D levels. Therefore, the relationship between apolipoprotein E (ApoE) and serum 25(OH)D warrants further investigation through approaches such as molecular mechanism studies (e.g., exploring isoform-specific effects of ApoE ϵ2, ϵ3, and ϵ4 alleles on vitamin D metabolism), Mendelian randomization (to address confounding biases), and large-scale epidemiological surveys (to minimize measurement errors and enhance statistical power). Additionally, integrating multi-omics data (e.g., transcriptomics or metabolomics) and longitudinal study designs could help clarify causality and dynamic interactions between ApoE, lipid profiles, and vitamin D status across diverse populations. Due to the cross-sectional nature of our research and resource constraints, we were unable to perform ApoE genotyping.in subsequent research, we will further explore the association between ApoE and vitamin D by investigating ApoE gene polymorphisms in conjunction with serological ApoE levels.

Research on the relationship between serum creatinine and 25(OH)D has predominantly focused on populations with renal dysfunction, particularly investigating associations with plasma clearance rates of 25(OH)D (32–34). There is a lack of direct studies examining the correlation between 25(OH)D and serum creatinine in individuals with normal renal function. This study suggest a positive correlation between the two, which may be attributed to elevated serum creatinine levels indicating reduced renal function. Notably, participants had normal renal function (serum creatinine within reference ranges), implying that the correlation may reflect mechanisms beyond renal clearance, such as age-related glomerular changes or shared inflammatory pathways. These observations necessitate large-scale epidemiological investigations to further validate this relationship and clarify its underlying mechanisms in broader populations.

Similar to previous results (35–37), this study also revealed an inverse correlation between age and serum 25(OH)D levels in older patients, which may be linked to age-related calcium loss and reduced cutaneous synthesis of vitamin D, and indicating that older adults should promptly supplement vitamin D to combat rising parathyroid hormone (PTH) (37).

This study was conducted over a multi-year recruitment period. Although vitamin D measurements were standardized to the same timeframe each year, interannual seasonal fluctuations can still affect vitamin D synthesis via sunlight exposure. Additionally, interyear differences in environmental factors (e.g., air quality and temperature) may influence participants’ outdoor activity levels, thereby impacting their vitamin D status. These factors introduce potential confounding variables to the results, representing a key limitation of this research.

In conclusion, this study provides evidence that lower serum 25(OH)D levels are associated with AMD presence and severity, particularly in late-stage disease. The novel association with ApoE highlights a potential link between lipid metabolism and vitamin D homeostasis, offering new insights into the complex pathophysiology of AMD. Additionally, significant correlations were observed between serum 25(OH)D levels and factors, including serum creatinine (Cr) and age, further emphasizing the multifactorial nature of vitamin D regulation in AMD. While 25(OH)D shows moderate promise as a diagnostic biomarker, its role in stage differentiation remains unclear. Future research should focus on longitudinal assessments, genetic polymorphisms, and mechanistic studies to validate these findings and explore vitamin D’s therapeutic potential in AMD prevention and management.

## Data Availability

The raw data supporting the conclusions of this article will be made available by the authors, without undue reservation.
